# Relationship between bone mineral density changes with denosumab treatment and risk reduction for vertebral and nonvertebral fractures

**DOI:** 10.1002/jbmr.1472

**Published:** 2012-03

**Authors:** Matthew Austin, Yu-Ching Yang, Eric Vittinghoff, Silvano Adami, Steven Boonen, Douglas C Bauer, Gerolamo Bianchi, Michael A Bolognese, Claus Christiansen, Richard Eastell, Andreas Grauer, Federico Hawkins, David L Kendler, Beatriz Oliveri, Michael R McClung, Ian R Reid, Ethel S Siris, Jose Zanchetta, Cristiano AF Zerbini, Cesar Libanati, Steven R Cummings

**Affiliations:** 1 Amgen Inc., Thousand OaksCA, USA; 2 UCSFSan Francisco, CA, USA; 3 University of VeronaVerona, Italy; 4 Leuven University, Division of Geriatric MedicineLeuven, Belgium; 5 Azienda Sanitaria GenoveseGenoa, Italy; 6 Bethesda Health Research CenterBethesda, MD, USA; 7 Center for Clinical and Basic ResearchBallerup, Denmark; 8 University of SheffieldSheffield, United Kingdom; 9 Hospital Universitario 12 de OctubreMadrid, Spain; 10 University of British ColumbiaVancouver, BC, Canada; 11 Sección Osteopatías Médicas, Hospital de Clínicas, Universidad de Buenos AiresBuenos Aires, Argentina; 12 Oregon Osteoporosis CenterPortland, OR, USA; 13 University of AucklandAuckland, New Zealand; 14 Columbia University Medical CenterNew York, NY, USA; 15 Instituto de Investigaciones Metabolicas and University of SalvadorBuenos Aires, Argentina; 16 Centro Paulista de Investigação ClinicaSão Paulo, Brazil; 17 San Francisco Coordinating Center, CPMC Research Institute, and UCSFSan Francisco, CA, USA

**Keywords:** DENOSUMAB, BONE MINERAL DENSITY, FRACTURE, SURROGATE, PERCENT OF TREATMENT EFFECT EXPLAINED

## Abstract

Dual-energy X-ray absorptiometric bone mineral density (DXA BMD) is a strong predictor of fracture risk in untreated patients. However, previous patient-level studies suggest that BMD changes explain little of the fracture risk reduction observed with osteoporosis treatment. We investigated the relevance of DXA BMD changes as a predictor for fracture risk reduction using data from the FREEDOM trial, which randomly assigned placebo or denosumab 60 mg every 6 months to 7808 women aged 60 to 90 years with a spine or total hip BMD *T*-score < −2.5 and not < −4.0. We took a standard approach to estimate the percent of treatment effect explained using percent changes in BMD at a single visit (months 12, 24, or 36). We also applied a novel approach using estimated percent changes in BMD from baseline at the time of fracture occurrence (time-dependent models). Denosumab significantly increased total hip BMD by 3.2%, 4.4%, and 5.0% at 12, 24, and 36 months, respectively. Denosumab decreased the risk of new vertebral fractures by 68% (*p* < 0.0001) and nonvertebral fracture by 20% (*p* = 0.01) over 36 months. Regardless of the method used, the change in total hip BMD explained a considerable proportion of the effect of denosumab in reducing new or worsening vertebral fracture risk (35% [95% confidence interval (CI): 20%–61%] and 51% [95% CI: 39%–66%] accounted for by percent change at month 36 and change in time-dependent BMD, respectively) and explained a considerable amount of the reduction in nonvertebral fracture risk (87% [95% CI: 35% – >100%] and 72% [95% CI: 24% – >100%], respectively). Previous patient-level studies may have underestimated the strength of the relationship between BMD change and the effect of treatment on fracture risk or this relationship may be unique to denosumab. © 2012 American Society for Bone and Mineral Research

## Introduction

Fractures are the main complication of osteoporosis and the goal of therapy is to reduce fracture risk. Bone mineral density (BMD) as assessed by dual energy x-ray absorptiometry (DXA) remains the most widely utilized measure to identify patients at risk for fracture. Epidemiological evidence demonstrates a strong relationship between decreases in BMD and increases in fracture risk.^1^ However, the relationship between gains in BMD and reduction in fracture risk in response to therapeutic intervention remains a topic of investigation. The relationship between treatment-induced BMD changes and fracture risk reduction has been reported based on individual patient-level clinical trial data,^2^–^10^ and on summary statistics from clinical trials using meta-analysis techniques.^5^, ^11^–^13^ At the study level, a robust relationship has been suggested. However, at the individual patient level, BMD changes with existing therapies appear to account for little of the fracture risk reduction observed, suggesting that BMD, although a strong predictor of fracture risk in untreated patients, is not a strong predictor for effects of osteoporosis treatments on fracture risk. These observations have brought into question the utility of serial BMD measurements to assess the effectiveness of osteoporosis therapy.^14^

Denosumab (Prolia^TM^) is a fully human monoclonal antibody against RANKL, a cytokine that is essential for the formation, function, and survival of osteoclasts.^15^, ^16^ It has been approved for the treatment of postmenopausal women with osteoporosis at increased risk for fracture. Denosumab results in a rapid and marked reduction in bone resorption, increases in BMD in the trabecular and cortical compartments, and significant reductions in fracture risk.^17^, ^18^ Denosumab results in an effect on BMD that is larger than that of the bisphosphonate alendronate.^19^–^22^ Of particular note is the positive impact of denosumab on the cortical skeleton.^21^, ^23^

In the FREEDOM trial, denosumab reduced the risk of new vertebral, hip, and nonvertebral fracture by 68% (*p* < 0.001), 40% (*p* = 0.04), and 20% (*p* = 0.01), respectively.^17^ Our goal was to estimate the proportion of the reduction in the risk of new or worsening vertebral and nonvertebral fracture with denosumab treatment that would be accounted for by changes in total hip BMD (percent of treatment effect explained). Both a standard analysis, based on BMD change at a fixed time point, and a more novel, time-dependent analysis were used.

## Methods

### Study design and subjects

The design of the FREEDOM trial has been reported previously^17^ and is summarized here. FREEDOM was a multinational, randomized, double-blind trial conducted at 214 centers in postmenopausal women (*N* = 7808) with a BMD *T*-score < –2.5 at the lumbar spine or total hip and not < –4.0 at either site. Subjects received either 60 mg denosumab or placebo subcutaneously every 6 months, and all subjects received daily supplements of calcium (≥1000 mg) and vitamin D (≥400 IU).

### Measurements

Yearly hip DXA BMD measurements were obtained for all subjects. New or worsening vertebral fractures were radiographically assessed at 12, 24, and 36 months per protocol. Additionally, if a subject presented with back pain suggestive of a vertebral fracture, an unscheduled X-ray was obtained and used to confirm a new or worsening vertebral fracture. Nonvertebral fractures were confirmed by imaging. All nonvertebral fractures with the exception of those of the skull, face, mandible, metacarpals, fingers, or toes were included in the analysis. Pathologic fractures and severe trauma fractures were not included. BMD and fracture assessments were performed by a central reader (Synarc, San Francisco, CA, USA) blinded to treatment assignment. This central vendor ensured quality control across centers and longitudinally on study.

### Statistical analyses

Historically,^2^–^6^, ^8^, ^10^, ^24^ the endpoint BMD (eg, BMD change at 12 or 36 months) has been used as the measure of BMD change when describing the relationship between change in BMD on therapy and fracture risk. In this traditional approach, the endpoint BMD change utilizes the BMD change at a fixed time point during the trial, even when the fracture may have occurred years before the BMD was measured (eg, the percent change in BMD being measured at 36 months and a fracture occurring 1 month after initiating therapy). To quantify the relationship between BMD and new or worsening vertebral fracture, a logistic regression model was used with new or worsening vertebral fracture during the study as the response, and randomized treatment and total hip BMD percent change from baseline at endpoint as covariates. Separate models were fitted for percent changes at 12, 24, and 36 months. When a BMD measure was not available at the time point of interest, the last available BMD measure (last observation carried forward) before that time point was used. Similar methods were employed for nonvertebral fracture using Cox's proportional hazards model.

In addition to this traditional approach, we explored the relationship when BMD was represented in a time-dependent manner. For the assessment of new or worsening vertebral fractures, a repeated-measure logistic regression was performed with treatment as a fixed effect and the BMD percent change from baseline as a time-dependent covariate. The annual BMD value (months 12, 24, and 36) was used with the corresponding annual vertebral fracture assessment. This analysis used new or worsening vertebral fractures and allowed for a subject to have a fracture at multiple time points.

When examining the relationship between time-dependent BMD changes and nonvertebral fractures, a repeated-measures model^25^ was used to estimate individual BMD on the actual BMD (g/cm^2^) scale at each unique nonvertebral fracture time. To accommodate expected nonlinearity in BMD changes, quadratic trajectories were fitted, with corresponding subject-specific random linear and quadratic coefficients, as well as random intercept. These estimates were converted to percent change from baseline, which was used to represent the specific total hip BMD at which each subject was at risk at the time of each fracture. Cox's proportional hazards model was then fitted with time-to-nonvertebral fracture as the response and randomized treatment and total hip time-dependent BMD percent change as covariates. Time-dependent BMD was treated as a time-dependent variable.^26^

For all models, the interaction between BMD changes and treatment was assessed. If the interaction was not significant at the 10% level, summaries of the model excluded the interaction term. A potential nonlinear relationship between fracture risk and change in BMD was explored through restricted cubic splines^27^ and/or quadratic polynomials.

The percent of treatment effect explained was used to help quantify these relationships, using Li's method^6^ for the point estimate and the delta method to estimate the confidence interval.^28^ The percent of treatment effect explained provides a measure for the extent to which the changes in BMD explain the observed reduction in fracture. If the percent of treatment effect explained is 100%, this indicates that all of the fracture risk reduction is explained through the change in BMD. Li's method does not guarantee that the point estimate and confidence interval for the percent of treatment effect are between 0% and 100%. The method involves fitting a statistical model that includes both the percent change in BMD and the treatment effect. The confidence interval will exceed 100% if the treatment effect in this model is not statistically significant. This can happen if the effect of treatment after adjusting for BMD changes is very small or nonexistent; or when there is a substantial treatment effect after adjusting for BMD changes, but the estimate of the treatment effect has low precision.

## Results

### Baseline characteristics

The baseline characteristics of the 7808 women randomized in the FREEDOM trial have been previously reported.^17^ Table 1 lists the relevant baseline characteristics for the current analysis. Age, total hip, and lumbar spine BMD *T*-score, prevalent vertebral fractures, and history of nonvertebral fracture were similar between the placebo and denosumab groups.

**Table 1:** Baseline Characteristics

	Placebo (*N* = 3906)	Denosumab (*N* = 3902)
Age (years), mean ± SD	72.3 ± 5.2	72.3 ± 5.2
Total hip BMD *T*-score, mean ± SD	–1.91 ± 0.81	–1.89 ± 0.81
Lumbar spine BMD *T*-score, mean ± SD	–2.84 ± 0.69	–2.82 ± 0.70
Prevalent vertebral fracture, % (*n*)	23.4% (915)	23.8% (929)
History of nonvertebral fracture in those ≥55 years, % (*n*)	30.1% (1177)	29.8% (1163)
History of nonvertebral fracture, % (*n*)	38.6% (1507)	39.1% (1524)

### Effect of denosumab on BMD and fractures at 12, 24, and 36 months

In the primary analysis of the FREEDOM trial, denosumab was associated with higher BMD changes at the total hip at each annual BMD assessment (Table 2). Additionally, denosumab significantly reduced the risk of new vertebral fractures at each annual assessment and of nonvertebral fractures at 24 and 36 months.

**Table 2:** Denosumab Treatment Effect on Total Hip BMD, and New or Worsening Vertebral and Nonvertebral Fracture Risk at 12, 24, and 36 Months

	BMD		Fracture			
		
	Mean (CI)	Difference Mean (CI)	New or worsening vertebral *n*^a^	New or worsening vertebral RR (CI)	Nonvertebral *n*^b^	Nonvertebral HR (CI)
Month 12						
Placebo	0.0 (−0.1, 0.1)	3.3 (3.1, 3.4)	82	0.39 (0.26, 0.58)	117	0.84 (0.65, 1.11)
Denosumab	3.2 (3.1, 3.3)		32		99	
Month 24						
Placebo	−0.7 (−0.8, −0.6)	5.1 (4.9, 5.2)	183	0.29 (0.21, 0.39)	214	0.79 (0.64, 0.96)
Denosumab	4.4 (4.3, 4.5)		53		170	
Month 36						
Placebo	−1.4 (−1.5, −1.3)	6.4 (6.2, 6.6)	264	0.32 (0.26, 0.41)	293	0.80 (0.67, 0.95)
Denosumab	5.0 (4.9, 5.1)		86		238	

*n* = number of subjects with ≥1 fracture; CI = 95% confidence interval; RR = risk ratio; HR = hazard ratio.

a There were 3691 women in the placebo group and 3702 in the denosumab group who were evaluable for new or worsening vertebral fractures.

b There were 3906 women in the placebo group and and 3902 women in the denosumab group who were evaluable for nonvertebral fractures.Difference and ratios reference placebo.

### Relationship between change in total hip BMD and new or worsening vertebral fracture efficacy

The analysis of the relationship between on-study changes in BMD and fracture efficacy required both a baseline and at least one postbaseline BMD measurement. Additionally, the new or worsening vertebral fracture outcome required at least one postbaseline radiograph. There were 7195 subjects in FREEDOM (3590 placebo, 3605 denosumab) who had a baseline and at least one postbaseline total hip BMD and vertebral X-ray assessment. Of these, 339 subjects (258 placebo, 81 denosumab) experienced a new or worsening vertebral fracture on study.

**Fig. 1:**
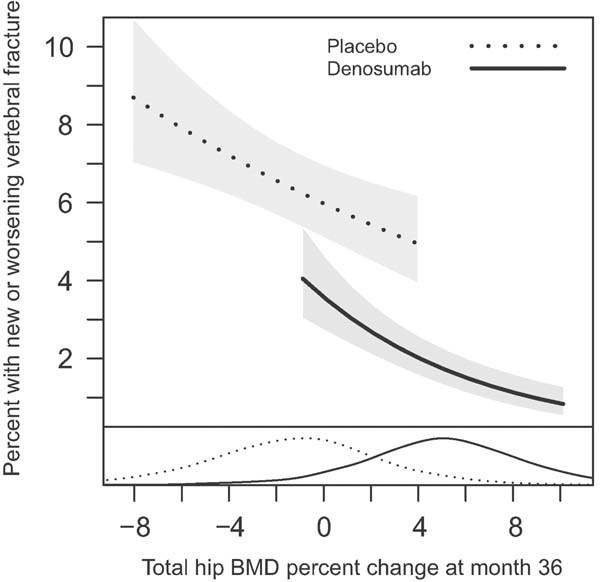
Relationship between new or worsening vertebral fracture incidence at 36 months and percent change from baseline in total hip BMD at 36 months. Adjusted estimates for a baseline lumbar spine BMD *T*-score of −2.5. Data represent the 5th through the 95th percentiles of total hip BMD percent change. The density curves at the bottom represent the distributions of total hip BMD change at 36 months for each treatment group. For both denosumab and placebo, the risk of new or worsening vertebral fracture decreased with increasing percent change in total hip BMD but the slope of the curves differed between treatment groups (interaction *p* value = 0.0003).

Figure 1 represents the relationship between percent change in total hip endpoint BMD at month 36 and new or worsening vertebral fracture risk. For both denosumab and placebo, the risk of fracture decreased with increasing percent change in total hip BMD but the slope of the curves differed between treatment groups (interaction *p* value = 0.0003). This relationship was further quantified through the percent of treatment effect explained. The percent change at month 36 in total hip endpoint BMD explained 35% (95% confidence interval [CI]: 20%–61%) of the treatment effect (Table 3). For the placebo and denosumab groups, each 1% increase in total hip BMD corresponded to a 4.9% and 13.5% reduction in new or worsening vertebral fracture risk, respectively.

**Table 3:** Summary of Percent of Treatment Effect Explained

	Percent of treatment effect explained	
	
Timing of BMD	New or worsening vertebral	Nonvertebral
12 months	23 (13, 40)	35 (9, >100^a^)
24 months	30 (16, 54)	89 (37, >100^a^)
36 months	35 (20, 61)	87 (35, >100^a^)
Time-dependent	51 (39, 66)	72 (24, >100^a^)

Values are % (95% confidence interval).

a Li's method allows for estimates of percent of treatment effect explained that exceed 100%. Estimates were truncated at 100%.

Assessment of the relationship between time-dependent BMD changes and new or worsening vertebral fractures also showed a decreasing risk of fracture with increasing BMD gains. Compared with no change from baseline, subjects with increased BMD had a lower risk of fracture and those with decreased BMD had a higher risk. The change in total hip time-dependent BMD explained 51% (95% CI: 39%–66%) of the new or worsening vertebral fracture risk reduction (Table 3). For the placebo and denosumab groups, each 1% increase in total hip BMD corresponded to a 9.4% and 14.5% reduction in new or worsening vertebral fracture risk, respectively.

### Relationship between change in total hip BMD and nonvertebral fracture efficacy

There were 7232 subjects in FREEDOM (3608 placebo, 3624 denosumab) who had a baseline and at least one postbaseline BMD assessment. Of these, 500 subjects (278 placebo, 222 denosumab) experienced a nonvertebral fracture on study.

**Fig. 2:**
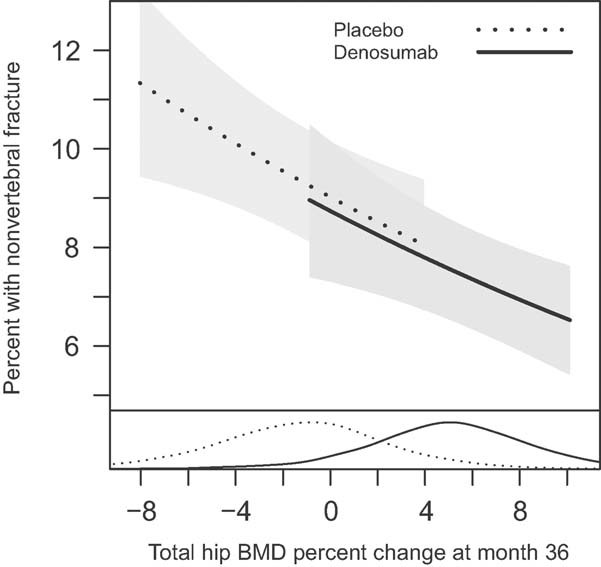
Relationship between nonvertebral fracture incidence at 36 months and percent change from baseline in total hip BMD at 36 months. Adjusted estimates for a baseline total hip BMD *T*-score of −2.5. Data represent the 5th through the 95th percentiles of total hip BMD percent change. The density curves at the bottom represent the distributions of total hip BMD change at 36 months for each treatment group. For both denosumab and placebo, the risk of nonvertebral fracture decreased with increasing percent change in total hip BMD. The data suggest similar relationships (slopes) for both treatment groups (interaction *p* value = 0.38).

Figure 2 represents the relationship between percent change in total hip BMD at month 36 and the incidence of nonvertebral fractures. For both denosumab and placebo, the risk of fracture decreased with increasing percent change in total hip BMD. The data suggest similar relationships (slopes) for both treatment groups (interaction *p* value = 0.38). After accounting for the effect of the percent change in total hip BMD, the treatment effect was no longer significant (*p* value = 0.97). The change in total hip BMD at month 36 explained 87% (95% CI: 35% to >100%) of the treatment effect (Table 3). A 1% change in total hip BMD at 36 months corresponded to a 3% change in nonvertebral fracture risk regardless of treatment. The majority of denosumab-treated patients showed positive changes and the majority of placebo-treated patients showed negative changes in total hip BMD.

Assessment of the relationship between time-dependent BMD changes and nonvertebral fractures also showed a decreasing risk of fracture with increasing BMD changes. Compared with no change from baseline, subjects with BMD gains had a lower risk of fracture and those with BMD losses had a higher risk. The change in total hip time-dependent BMD explained 72% (95% CI: 24% to >100%) of the treatment effect (Table 3). A 1% total hip BMD increase corresponded to a 4.5% reduction in nonvertebral fracture risk.

## Discussion

A strong relationship between DXA BMD and fracture risk has been demonstrated by a number of epidemiological studies.^1^ However, the relationship between increases in BMD and reduction in fracture risk as a result of therapeutic intervention is less well established. Our analyses showed that with denosumab treatment, larger increases in total hip DXA BMD were related to greater reductions in new or worsening vertebral and nonvertebral fracture risk. Regardless of the methodology used (fixed time point or time-dependent models), the change in total hip BMD may explain a considerable proportion (35%–51%) of the effect of denosumab on risk reduction of new or worsening vertebral fractures and appears to explain a considerable amount of the reduction in risk of nonvertebral fracture (∼80%).

The relationship between BMD change and fracture risk effect in women treated for osteoporosis has been the subject of previous reports. At the study level, a robust relationship has been suggested. However, at the patient level, the relationship has shown limited contributions of the BMD change to the reduction in fracture risk. Hochberg et al.^24^ showed that larger increases in total hip and/or spine BMD while on alendronate therapy were associated with lower risk of new vertebral fractures. Cummings et al.^5^ reported that in women with a prevalent vertebral fracture, larger increases in spine BMD at 12 months were associated with lower fracture risk with alendronate; however, BMD changes only accounted for 16% (95% CI: 11%–27%) of the new vertebral fracture risk reduction. Li et al.^6^ showed that the BMD changes with 5.0 mg daily risedronate from the VERT-NA and VERT-MN trials accounted for 28% (95% CI: 16%–49%) of the fracture risk reduction. Additionally, Li et al.^6^ showed that the treatment effect was still significant at the 5% level after adjusting for the BMD changes. Watts et al.^9^ combined the VERT-NA, VERT-MN, and HIP trials to report that for risedronate, positive changes in lumbar spine and femoral neck BMD over 3 years were similar with respect to fracture reduction regardless of magnitude, and changes in BMD explained only 18% (95% CI: 10%–26%) or 11% (95% CI: 7%–15%) of vertebral fracture efficacy, respectively. In a separate study, Watts et al.^10^ showed for risedronate that 3-year changes in lumbar spine and femoral neck BMD explained only 12% (95% CI: 2%–21%) and 7% (95% CI: 2%–13%), respectively, of nonvertebral fracture efficacy. Wasnich et al.^8^ showed that for ibandronate, total hip BMD change at year 3 was a significant predictor of vertebral fracture risk reduction, reporting that a 1% increase in total hip BMD accounted for a 7.9% (*p* = 0.0084) reduction in risk. Sarkar et al.^7^ reported that femoral neck BMD changes at 1 and 3 years were related to the risk of new vertebral fracture for both raloxifene and placebo; however, a significant treatment effect remained after adjusting for BMD changes. Changes in femoral neck BMD at 3 years accounted for only 4% of the vertebral fracture risk reduction. Changes in lumbar spine BMD at 3 years was not associated with new vertebral fracture risk in the raloxifene group, but was negatively correlated with fracture risk in the placebo group.

The relationship, including slopes and intercepts, between total hip BMD and nonvertebral fracture risk in the current study was similar for those patients on placebo, most of whom lost BMD, and those treated with denosumab, most of whom gained BMD during the 3-year FREEDOM trial (Fig. 2). Because DXA BMD assessment is influenced by (but does not distinguish between) bone geometry and mineral content of the trabecular and cortical compartments, it can be hypothesized that therapies that influence those compartments in a different proportion than occurs during the bone loss process would not result in changes in density that conserve the relationship between DXA BMD and biomechanical strength and fracture risk. For example, if steel were removed proportionally from both the suspender cables and the pillars of a bridge, the resulting decrease in strength would not be corrected by replacing the total removed amount of steel just to the suspender cables and not the pillars. The relationship between DXA BMD change and fracture risk observed with denosumab in our study may be explained by the reported positive effect of denosumab not only on the trabecular but also on the cortical compartment. Indeed, Seeman et al.^21^ reported differences in cortical BMD and thickness with denosumab compared with alendronate therapy and demonstrated that these differences impacted polar moment of inertia estimates at the radius and tibia. In addition, changes from baseline and from placebo at both the trabecular and the cortical hip compartments have been reported using QCT scans from a subset of subjects in the FREEDOM trial and these improvements resulted in increases in estimated failure load as determined by finite element analysis.^29^, ^30^ Altogether, these results suggest that the distribution in bone density gains within the cortical and trabecular compartments with denosumab treatment may be different than the distribution achieved with other therapies and may contribute to the different relationships observed between BMD gains and fracture risk. Another possible explanation for the difference between these findings and those of other therapies could be the size of the study and the number of events observed, which allowed for a more precise estimate of the relationship between BMD and fracture.

This study has several strengths: it involved a large number of patients with baseline and follow-up assessments, it utilized individual subject data, and it evaluated the relationship between BMD changes and fracture risk using a time-dependent analysis in addition to the standard endpoint methodological approach previously used by others. The time-dependent analysis had the advantage of using an estimated BMD at the time of the actual fracture event instead of relating a change in BMD at previous or later time points. Importantly, regardless of the approach, the results obtained were similar.

Limitations of the study include the fact that no active comparator data were obtained in the same trial, that the study only enrolled untreated patients at baseline, and that only hip BMD was measured annually in all subjects. As a result, direct comparisons of our results to those of other studies cannot be made, results for lumbar spine DXA BMD may differ, and it is not known if these relationships apply to the BMD gains observed with denosumab in subjects previously treated with alendronate.^20^ Additionally, the precision for the estimate of the percent of treatment effect explained is low, leading to large confidence intervals. Although fracture studies are powered to detect treatment differences, they are generally underpowered for the assessment of surrogate biomarkers in individual studies. Furthermore, the analyses in this study included all fractures and did not exclude fractures that occurred before the measurement of the endpoint BMD as in previous analyses. The assessment of change in BMD after fracture could be impacted by the loss of mobility or increased bed rest after fracture, which could potentially bias assessment of the relationship between changes in BMD and fracture risk.

In summary, we found that gains in total hip BMD explain a considerable proportion of the fracture risk reductions observed with denosumab. Previous studies may have underestimated the value of change in DXA BMD as a surrogate marker for the effect of treatment on fracture risk or the relationship may be unique to denosumab.

## Disclosures

This study was funded by Amgen Inc. MA, Y-CY, AG, and CL are Amgen employees and own Amgen stock and/or stock options. EV has nothing to disclose. SA has received consulting fees from Servier, Novartis, Amgen, Roche, and Eli Lilly. He has also received lecture fees from Novartis, Amgen, Roche, and Servier. SB has received funding for serving as a trial investigator and a member of a steering committee for Amgen and has received consulting fees from Amgen. DB has received research support from Novartis and Amgen. GB has served on an advisory committee and/or speaker's bureau for Abbott, MSD, Novartis, Roche, Servier, and Amgen. He has also received consulting fees from Schering Plough and Pfizer. MAB has received research grants from Eli Lilly, Amgen, and Sanofi Aventis. He has also served on the speaker's bureau for Eli Lilly, Amgen, and Novartis. CC is the chairman of Nordic Bioscience A/S and of CCBR/Synarc. He has also received consulting fees from Roche, Wyeth-Ayerst, Eli Lilly, Novartis, Novo Nordisk, Procter & Gamble, Groupe Fournier, Besins EscoVesco, MSD, Chiesi, Boehringer Mannheim, Pfizer, and Amgen. RE serves as a consultant, has received honoraria for speaking, and has received grant support from Amgen, AstraZeneca, California Pacific Medical Center, GlaxoSmithKline, Hologic, Kyphon Inc., Lilly Industries, Maxygen, Nastech Pharmaceuticals, Nestle Research Center, New Zealand Milk Limited, Novartis, Novo Nordisk, ONO-Pharma, Organon Laboratories, Osteologix, Pfizer, Procter & Gamble Pharmaceuticals, Roche Diagnostics, Sanofi-Aventis, Servier, Shire, Tethys, TransPharma Medical Limited, Unilever, and Unipath. FH has nothing to disclose. DLK has received research grants from Merck, Boehringer, J&J, Eli Lilly, GSK, Servier, Pfizer, Amgen, Novartis, and Biosante. He has also received consulting fees from Merck, Eli Lilly, Pfizer, Amgen, and Novartis and has served on a speaker's bureau for Eli Lilly, Pfizer, Amgen, and Novartis. BO has nothing to disclose. MRM has received funding for serving as a trial investigator and a member of a steering committee for Amgen, and has received consulting and lecture fees from Amgen. He has also received research grants, consulting fees and/or lectures fees from Lilly, Merck, Novartis, Takeda, and Warner-Chilcott. IRR has received research grants or consultancy fees from Amgen, Novartis, Procter & Gamble, and Merck. ESS is a consultant, advisory board member, and/or speaker for Amgen, Eli Lilly, Merck, Novartis, and Pfizer. JZ has received consulting fees from Amgen, Eli Lilly, Pfizer, Merck, Servier, and GSK. CZ has nothing to disclose. SRC has received consulting fees from Amgen.
